# Special Issue “Cancer Immunotherapy: Tumor Microenvironment, Biomarker Discovery and Immune Resistance”

**DOI:** 10.3390/ijms25105113

**Published:** 2024-05-08

**Authors:** Jason Yongsheng Chan

**Affiliations:** 1 Division of Medical Oncology, National Cancer Centre Singapore, Singapore 168583, Singapore; jason.chan.y.s@singhealth.com.sg; Tel.: +65-6436-8000; Fax: +65-6227-2759; 2 Cancer Discovery Hub, National Cancer Centre Singapore, Singapore 168583, Singapore

Since the launch of this Special Issue entitled “Cancer Immunotherapy: Tumor Microenvironment, Biomarker Discovery and Immune Resistance”, the field of cancer immunotherapy has continued to witness rapid growth in the development of novel agents, improvements in our understanding of mechanisms of response and resistance, and the maturation of emerging technologies such as artificial intelligence, machine learning, single-cell sequencing and spatial profiling [1]. Although a key unmet need continues to exist in that treatment resistance occurs in the vast majority of patients, we are perhaps now at an inflection point, with new opportunities to overcome challenges and limitations in identifying predictive biomarkers for clinical deployment.

Immunotherapy is now firmly established as the fifth pillar of cancer therapeutics, joining the armamentarium alongside surgery, radiation, chemotherapy and targeted therapy. In particular, immune checkpoint inhibitors and chimeric antigen receptor (CAR) T-cell therapies have been brought to the forefront of cancer treatment across a wide spectrum of indications, often providing not just additional clinical benefits but also the chance of a cure [2,3]. Despite the remarkable progress made, predicting which patients respond to immunotherapy remains a difficult task, and treatment resistance—either primary or adaptive—is commonly observed. Biologically, resistance to immunotherapy can be attributed to both factors intrinsic to tumor cells and extrinsic factors related to the tumor microenvironment [4].

The tumor microenvironment comprises a complex interplay of biological interactions contributing to known hallmarks of cancer, including features such as sustained angiogenesis, deregulated cellular metabolism, and immune evasion [5]. Collectively, these influence immune cell infiltration, accumulation and activation, thereby modulating immunotherapy response. In hematological malignancies including lymphoma, leukemia and multiple myeloma, an immunosuppressive milieu in the tumor microenvironment can negatively impact CAR-T cell efficacy outcomes (reviewed in [6]). A recent report suggested that tumor gene expression signatures representative of immune contextures, as derived using the NanoString PanCancer IO360 panel, correlated significantly with CAR-T therapy outcomes. Specifically, B-cell and stromal immunosuppressive signatures were associated with better and worse outcomes, respectively, in relation to second-line axicabtagene ciloleucel (axi-cel) therapy in large B cell lymphoma [7]. Other interesting tumor microenvironmental features of recent interest include tertiary lymphoid structures (TLS), which are lymph node-like cell clusters highly enriched in B-cells and plasma cells. These structures have been suggested to confer a favorable response to immune checkpoint blockade in various cancer types. Notably, a phase II multicenter trial (REGOMUNE) evaluated the combination of the PD-L1 inhibitor avelumab with regorafenib in advanced solid tumors harboring mature TLS, demonstrating clinical benefits in 16 of 34 patients (47%) [8]. Our level of understanding of the landscape of tumor-infiltrating T-lymphocytes has also continued to evolve. Chu et al. investigated single-cell sequences of 308,048 transcriptomes across 16 cancer types to generate an atlas of tumor-infiltrating T-cells, revealing heterogeneous cellular subpopulations and identifying a stress response state characterized by significantly upregulated heat shock gene expression. Such a stress response was observed in intratumoral CD4/CD8+ cells following immune checkpoint inhibition, particularly in nonresponsive tumors, suggesting a potential role in immunotherapy resistance [9]. Collectively, these data imply that a deeper appreciation of the intricacies of various components of the tumor and its microenvironment will be necessary in order to better predict treatment response and prognostic outcomes in relation to the various immunotherapies.

Beyond the tumor microenvironment, other host-related systemic factors add to the complexity of understanding immunotherapy response and resistance. For example, the gut microbiome, host immune response, hormonal factors, and neuronal signals may directly or indirectly influence the tumor cells and/or their surrounding microenvironment. Neuronal signals, such as β2-adrenergic receptor-mediated signaling, mediate immune cell changes and thereby play an important role in regulating the tumor microenvironment in the form of a neuroimmune axis [4]. The composition of the intestinal microbiota, referred to as Gut OncoMicrobiome signatures (GOMS), as derived for example via shotgun metagenomic sequencing of stool samples, may predict responses or resistance to immune checkpoint inhibitors [10].

State-of-the-art technologies are currently enabling multi-dimensional, high-throughput and hyperplex profiling of the tumor microenvironment in tissue specimens. These include spatial transcriptomics and proteomics methods such as NanoString digital spatial profiling (DSP), BGI Stereo-seq, Vizgen MERSCOPE and 10X Visium [1]. These innovations are increasingly being combined with artificial intelligence and deep learning algorithms in data analysis [11], alongside a suite of computational immunogenomic approaches developed to interrogate both cancer-intrinsic features (e.g., tumor mutation burden, neoantigen burden, HLA genotype) and cancer-extrinsic features (e.g., tumor microenvironmental cellular position, cell–cell communication networks, T- and B-cell repertoire diversity) which may predict treatment response [12]. These novel methodologies will hopefully not just drive the discovery of robust predictive biomarkers for existing therapies but also accelerate the identification of new therapeutic targets. In conclusion, as we stand at this juncture of heightened understanding and technological progress, collaborative efforts across disciplines are poised to drive forward a new era of precision immuno-oncology, bringing us closer to more effective and personalized cancer treatments.
